# Five-year follow-up results from phase II studies of nivolumab in Japanese patients with previously treated advanced non-small cell lung cancer: pooled analysis of the ONO-4538-05 and ONO-4538-06 studies

**DOI:** 10.1093/jjco/hyaa157

**Published:** 2020-10-06

**Authors:** Hideo Saka, Makoto Nishio, Toyoaki Hida, Kazuhiko Nakagawa, Hiroshi Sakai, Naoyuki Nogami, Shinji Atagi, Toshiaki Takahashi, Hidehito Horinouchi, Mitsuhiro Takenoyama, Nobuyuki Katakami, Hiroshi Tanaka, Koji Takeda, Miyako Satouchi, Hiroshi Isobe, Makoto Maemondo, Koichi Goto, Tomonori Hirashima, Koichi Minato, Nobumichi Yada, Tomohide Tamura

**Affiliations:** Department of Medical Oncology, Nagoya Medical Center, Nagoya; Department of Respiratory Medicine, Matsunami General Hospital, Gifu; Department of Thoracic Medical Oncology, Cancer Institute Hospital, Tokyo; Department of Thoracic Oncology, Aichi Cancer Center Hospital, Nagoya; Department of Medical Oncology, Faculty of Medicine, Kindai University, Osaka; Department of Thoracic Oncology, Saitama Cancer Center, Saitama; Division of Thoracic Oncology and Medicine, National Hospital Organization Shikoku Cancer Center, Ehime; Department of Thoracic Oncology, Kinki-Chuo Chest Medical Center, Sakai; Division of Thoracic Oncology, Shizuoka Cancer Center, Shizuoka; Department of Thoracic Oncology, National Cancer Center Hospital, Tokyo; Department of Thoracic Oncology, National Hospital Organization Kyushu Cancer Center, Fukuoka; Department of Medical Oncology, Kobe City Medical Center General Hospital, Kobe; Department of Internal Medicine, Niigata Cancer Center Hospital, Niigata, Japan; Department of Medical Oncology, Osaka City General Hospital, Osaka, Japan; Department of Thoracic Oncology, Hyogo Cancer Center, Hyogo, Japan; Department of Medical Oncology, KKR Sapporo Medical Center, Sapporo, Japan; Division of Pulmonary Medicine, Department of Internal Medicine, Iwate Medical University School of Medicine, Iwate, Japan; Department of Respiratory Medicine, Miyagi Cancer Center, Miyagi, Japan; Division of Thoracic Oncology, National Cancer Center Hospital East, Chiba, Japan; Department of Thoracic Oncology, Osaka Habikino Medical Center, Osaka, Japan; Division of Respiratory Medicine, Gunma Prefectural Cancer Center, Gunma, Japan; Medical Affairs, Ono Pharmaceutical Co., Ltd, Osaka, Japan; Thoracic Center, St. Luke’s International Hospital, Tokyo, Japan

**Keywords:** nivolumab, survival, non-small cell lung cancer, safety

## Abstract

**Background:**

Two phase II studies in Japan examined the efficacy and safety of nivolumab, a programmed cell death 1 receptor inhibitor, in patients with advanced squamous and non-squamous non-small cell lung cancer (ONO-4538-05 and ONO-4538-06). We examined the long-term efficacy and safety of nivolumab in these patients treated for up to 5 years.

**Methods:**

Patients with squamous (*N* = 35) or non-squamous (*N* = 76) non-small cell lung cancer received nivolumab (3 mg/kg every 2 weeks) until disease progression/death. Overall survival and progression-free survival were assessed at 5 years after starting treatment in separate and pooled analyses. Safety was evaluated in terms of treatment-related adverse events.

**Results:**

A total of 17 patients were alive at the database lock (26 July 2019). The median overall survival (95% confidence interval) and 5-year survival rate were 16.3 (12.4–25.2) months and 14.3% in squamous patients, 17.1 (13.3–23.0) months and 19.4% in non-squamous patients and 17.1 (14.2–20.6) months and 17.8% in the pooled analysis, respectively. Programmed death ligand-1 expression tended to be greater among 5-year survivors than in non-survivors (*P* = 0.0703). Overall survival prolonged with increasing programmed death ligand-1 expression, with 5-year survival rates of 11.8, 21.8 and 41.7% in patients with programmed death ligand-1 expression of <1, ≥1–<50 and ≥50%, respectively. Treatment-related adverse events in ≥10% of patients (pooled analysis) included rash (15.3%), malaise (14.4%), decreased appetite (14.4%), pyrexia (14.4%) and nausea (10.8%).

**Conclusions:**

Long-term survival with nivolumab was observed in patients with squamous or non-squamous non-small cell lung cancer. No new safety signals were reported after ≥5 years of follow-up.

## Teaser

This long-term follow-up demonstrated ongoing efficacy and safety of nivolumab over ≥5 years in patients with squamous or non-squamous non-small cell lung cancer in Japan.

## Introduction

Lung cancer is the leading cause of cancer-related deaths worldwide (1) and is associated with a significant burden to patients in terms of disability and years of life lost (2). Non-small cell lung cancer (NSCLC) is the predominant type of lung cancer, and the main histological subtypes are squamous (SQ) or non-squamous (non-SQ) NSCLC (3). For many years, docetaxel has been recommended as second-line therapy for patients who progress after first-line chemotherapy. Although various alternatives, including pemetrexed and epidermal growth factor receptor (*EGFR*) gene-tyrosine kinase inhibitors, may be used as second-line therapy, these drugs generally failed to show superior or non-inferior effects on overall survival (OS) relative to docetaxel in this setting (4).

Further understanding of the molecular biology and pathogenesis of NSCLC has led to the development of novel classes of anti-angiogenic drugs and immune checkpoint inhibitors (4). One immune checkpoint pathway involves the programmed cell death 1 (PD-1) receptor, a T-cell checkpoint receptor. A PD-1-mediated immune repressive signal is activated by programmed death ligand-1 (PD-L1), which is expressed on various cancers, including NSCLC (5–7). Accordingly, inhibiting PD-1 or PD-L1 enhances intratumoral immune responses (5).

Nivolumab is a fully human PD-1 antibody that disrupts PD-1-mediated signalling and has been approved for the treatment of patients with metastatic NSCLC and disease progression on or after platinum-based chemotherapy (8). The approval of nivolumab was driven by two international phase III trials (CheckMate 017 and CheckMate 057) in which nivolumab demonstrated superior OS and response rates compared with docetaxel in previously treated patients with advanced SQ (9) and non-SQ (10) NSCLC. In Japan, two multicentre phase II clinical trials investigated the efficacy and safety of nivolumab in patients with advanced or recurrent SQ (11) (ONO-4538-05) and non-SQ (12) (ONO-4538-06) NSCLC. In these studies, the overall response rates (ORR) were 25.7 and 22.4% in SQ and non-SQ patients, respectively. Considering that the prognosis of patients with NSCLC is generally poor, with relatively short OS, follow-up of patients enrolled in the ONO-4538-05 and ONO-4538-06 studies continued to monitor long-term outcomes.

We recently reported the 3-year survival rates of 20.0 and 31.9% for SQ and non-SQ patients, respectively. Of 111 patients enrolled in both studies, 30 survived for ≥3 years, eight of which were still receiving nivolumab (13). To provide further insight into the long-term effects of nivolumab in Japanese patients with SQ or non-SQ NSCLC, we have now assessed the 5-year OS of patients enrolled in the ONO-4538-05 and ONO-4538-06 studies.

## Methods

### Overview and ethics

The designs of ONO-4538-05 in SQ patients (11) and ONO-4538-06 in non-SQ patients (12) are reported in more detail in the prior publications. The present analyses were performed in a similar manner to our prior report on 3-year survival (13). Both studies adhered with the Declaration of Helsinki and relevant local/international guidelines. The studies were registered on the Japan Pharmaceutical Information Center-Clinical Trials Information database (JapicCTI-132072 and JapicCTI-132073). The studies were approved by institutional review boards at each participating site. All patients provided written informed consent.

### Patients

In brief, both studies enrolled patients aged ≥20 years, Eastern Cooperative Oncology Group performance status of 0 or 1 and stage IIIB/IV NSCLC with histological or cytological confirmation of SQ or non-SQ. Patients with recurrent SQ/non-SQ NSCLC after surgical resection were eligible.

### Study design and treatments

Both studies were multicentre, open-label, phase II studies in Japan. ONO-4538-05 enrolled 35 patients across 17 sites between May 2013 and April 2014. ONO-4538-06 enrolled 76 patients across 19 sites between April and October 2013. The cut-off date for 5-year survival was 26 July 2019. Patients received nivolumab at a dose of 3 mg/kg intravenously every 2 weeks in 6-week cycles. Treatment was stopped upon radiologic confirmation of progressive disease, onset of an unacceptable toxicity, withdrawal or death.

### PD-L1 analysis

Tumour PD-L1 expression was retrospectively assessed in pre-treatment (archival or recent) tumour biopsy specimens using a validated, automated immunohistochemical assay (Dako North America) with a rabbit anti-human PD-L1 antibody (clone 28–8, Epitomics). Tumour PD-L1 expression was confirmed when the tumour cell membranes were stained (at any intensity) in a section with ≥100 evaluable tumour cells.

**Figure 1.**
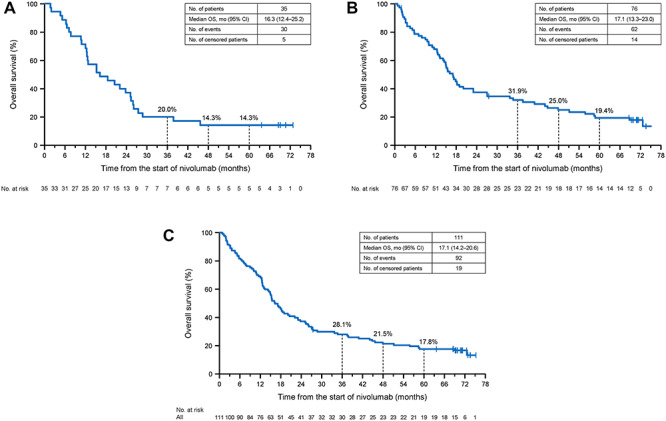
Kaplan–Meier plots of overall survival (OS) in patients with SQ NSCLC (A), non-SQ NSCLC (B) and in a pooled analysis of SQ and non-SQ NSCLC patients (C). NSCLC, non-small cell lung cancer; SQ, squamous.

### Endpoints

The primary endpoint in both studies was the ORR, which was assessed by an Independent Review Committee and was calculated as a best overall response of complete response (CR) or partial response (PR) defined by Response Evaluation Criteria in Solid Tumors guidelines version 1.1. Secondary endpoints included the disease control rate (DCR; sum of CR, PR or stable disease), duration of response (DOR), OS and progression-free survival (PFS). Safety was evaluated in terms of adverse events (AEs) graded according to the National Cancer Institute Common Terminology Criteria for AEs version 4.0. Tumour PD-L1 expression was assessed in pre-treatment tumour-biopsy specimens by immunohistochemistry, as described in the previous study (13).

### Statistical analyses

The sample sizes of both studies were calculated based on the threshold response rate for nivolumab, and the efficacy and safety outcomes were assessed in all patients who received at least one dose of nivolumab in each study (11,12). For the purpose of this report, we analysed the efficacy and safety data for SQ and non-SQ NSCLC patients separately and pooled together. The χ^2^ or Wilcoxon signed-rank tests were used to compare non-parametric variables at baseline. OS was calculated as the time (days) as follows: ‘date of death from any cause’ − ‘date of the first dose of nivolumab’ + 1. PFS was calculated as the time (days) as follows: ‘date of disease progression or death, whichever comes first’ − ‘date of the first dose of nivolumab’ + 1. OS and PFS curves were plotted using the Kaplan–Meier method, and the OS and PFS rates were calculated at 36, 48 and 60 months. We also compared OS and PFS according to tumour PD-L1 expression (cells with PD-L1 expression: <1, ≥1–<50 or ≥50%) in the pooled population and in non-SQ patients separately. AEs are reported as the number (percent) of patients with treatment-related AEs (TRAEs) according to SQ/non-SQ NSCLC type and in the pooled analysis.

## Results

### Patients and nivolumab administration

A total of 111 patients were enrolled and treated, 35 with SQ and 76 with non-SQ NSCLC. Among SQ NSCLC patients, the median duration of exposure to nivolumab and median number of doses of nivolumab were 110 days (range 15–2216 days) and 8 (range 2–152), respectively. The corresponding values in non-SQ NSCLC patients were 74.5 days (1–2290 days) and 6 (range 1–152). One SQ NSCLC patient and five non-SQ NSCLC patients were still receiving nivolumab at the data cut-off, with ongoing responses and administration for >60 months. A total of 17 patients were alive at the database lock (26 July 2019).

### ORR, DCR and DOR

In SQ NSCLC patients, ORR (central assessment), DCR and median DOR were 25.7% (95% CI 14.2–42.1%), 54.3% (95% CI 38.2–69.5%) and not reached (range 3.0–68.9 months), respectively. In non-SQ NSCLC patients, the ORR (central assessment), DCR and median DOR were 22.4% (95% CI 14.5–32.9%), 47.4% (95% CI 36.5–58.4%) and 41.5 months (range 1.6–72.1 months), respectively. In SQ and non-SQ NSCLC patients combined, ORR, DCR and median DOR were 23.4% (95% CI 16.5–32.1%), 49.5% (95% CI 40.4–58.7%) and 41.5 months (1.6–72.1 months), respectively.

### OS

**Figure 2.**
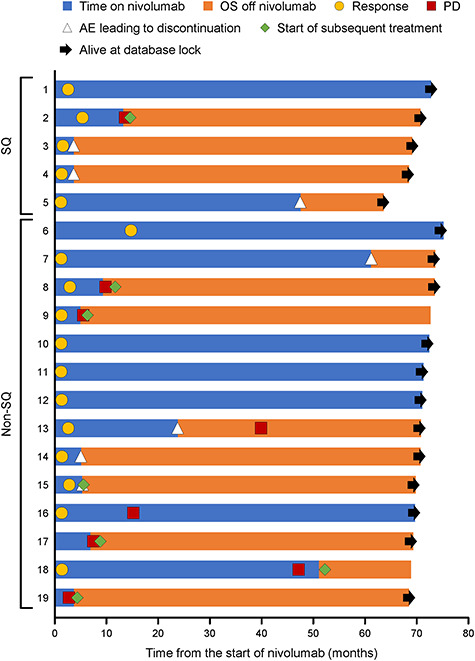
Swimmer plot of 5-year survivors. AE, adverse event; OS, overall survival; PD, disease progression; SQ, squamous.

The median OS in SQ NSCLC patients was 16.3 months (95% CI 12.4–25.2 months). The estimated 4- and 5-year survival rates were 14.3 (95% CI 5.2–27.7%) and 14.3% (95% CI 5.2–27.7%), respectively (Fig. 1A). The median OS in non-SQ NSCLC patients was 17.1 months (95% CI 13.3–23.0 months), with estimated 4- and 5-year survival rates of 25.0 (95% CI 15.8–35.2%) and 19.4% (95% CI 11.3–29.1%), respectively (Fig. 1B). In the pooled analysis of SQ and non-SQ NSCLC patients, the median OS was 17.1 months (95% CI 14.2–20.6 months) and the 4- and 5-year OS rates were 21.5 (95% CI 14.4–29.7%) and 17.8% (11.3–25.5%) (Fig. 1C). Among 111 NSCLC patients, 19 were still alive after 5 years. This included five SQ NSCLC patients and 14 non-SQ NSCLC patients. Of these 19 patients, 17 (89.5%) showed a response (CR or PR) to nivolumab (Fig. 2). Of 92 patients who died before 5 years, nine (9.8%) showed a response to nivolumab. In the pooled analysis, we found no marked differences in characteristics of survivors and non-survivors at 5 years (Table 1) except for a tendency towards higher PD-L1 expression among 5-year survivors than in non-survivors (*P* = 0.070).

### PFS

**Figure 3.**
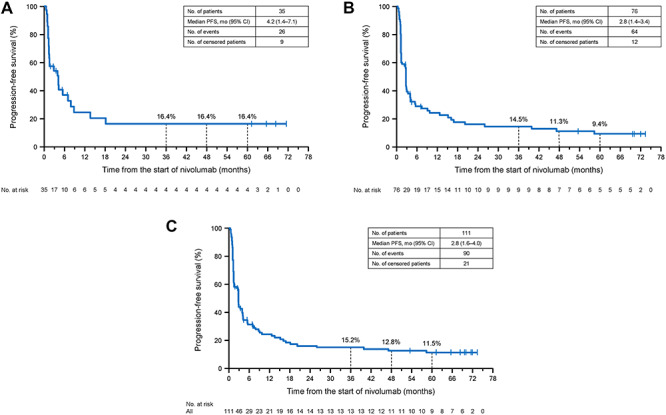
Kaplan–Meier plots of progression-free survival (PFS) in patients with SQ NSCLC (A), non-SQ NSCLC (B) and in a pooled analysis of SQ and non-SQ NSCLC patients (C). NSCLC, non-small cell lung cancer; SQ, squamous.

Centrally assessed PFS was determined in SQ NSCLC patients, non-SQ NSCLC patients and in the pooled population (Fig. 3). In SQ NSCLC patients, the median PFS was 4.2 months (95% CI 1.4–7.1 months), with estimated 4- and 5-year PFS rates of 16.4 (95% CI 5.4–32.5%) and 16.4% (95% CI 5.4–32.5%), respectively (Fig. 3A). In non-SQ NSCLC patients, the median PFS and the 4- and 5-year PFS rates were 2.8 months (95% CI 1.4–3.4 months), 11.3% (95% CI 5.1–20.3%) and 9.4% (95% CI 3.8–18.2%), respectively (Fig. 3B). In the pooled population, the median PFS was 2.8 months (95% CI 1.6–4.0 months) and the estimated 4- and 5-year PFS rates were 12.8 (95% CI 6.9–20.6%) and 11.5% (95% CI 6.0–19.1%), respectively (Fig. 3C).

**Table 1:** Patient characteristics

	5-year survivors (*N* = 19)	Non-survivors (*N* = 92)	*P* value^a^
Age (years)	64.0 (45.0–70.0)	65.0 (31.0–85.0)	0.33
Males	14 (73.7)	67 (72.8)	0.94
ECOG PS 0	10 (52.6)	36 (39.1)	0.28
Presence of brain metastases	3 (15.8)	21 (22.8)	0.50
One prior systemic regimen	17 (89.5)	73 (79.3)	0.30
Former/current smoker	16 (84.2)	73 (79.3)	0.63
*EGFR*m status, WT/unknown	17 (89.5)	72 (78.3)	0.26
PD-L1 expression level			
Available (*n*)	13	46	
<1%	2 (15.4)	15 (32.6)	0.070
≥1–<50%	6 (46.2)	24 (52.2)	
≥50%	5 (38.5)	7 (15.2)	

Values are median (range) or *n* (%).

ECOG PS, Eastern Cooperative Oncology Group performance status; *EGFR*m, epidermal growth factor receptor gene mutation; PD-L1, programmed death ligand-1; WT, wild type.

^a^Wilcoxon’s signed-rank test was used for statistical comparisons of age and PD-L1, and the χ^2^ test was used for all other statistical comparisons.

### Impact of PD-L1 expression on survival outcomes

PD-L1 expression was measurable in 59/111 patients and was classified as <1, ≥1–<50 and ≥50% in 17 (28.8%), 30 (50.8%) and 12 (20.3%) patients, respectively. OS increased in parallel with PD-L1 expression, with 5-year survival rates of 11.8, 21.8 and 41.7% in patients with PD-L1 expression of <1, ≥1–<50 and ≥50%, respectively (Supplementary Fig. 1a). PFS prolonged with increasing PD-L1 expression (Supplementary Fig. 1b). In non-SQ NSCLC patients, PD-L1 expression was measurable in 40/76 patients and was classified as <1, ≥1–<50 and ≥50% in 13 (32.5%), 20 (50.0%) and 7 (17.5%) patients, respectively. As in all patients combined, OS and PFS increased in parallel with PD-L1 expression in the non-SQ cohort (Supplementary Fig. 2a and b). The 5-year survival rates were 15.4, 22.5 and 57.1% in patients with PD-L1 expression of <1, ≥1–<50 and ≥50%, respectively. Due to the small number of SQ NSCLC patients, survival outcomes were not analysed according to PD-L1 expression in this group.

### Safety

TRAEs of any grade occurred in 68.6% of SQ NSCLC patients and 86.8% of non-SQ NSCLC patients, and included grade 3–4 TRAEs in 8.6 and 25.0% of patients, respectively (Table 2). Serious TRAEs occurred in 5.7 and 21.1% of SQ and non-SQ patients, respectively. TRAEs led to discontinuation of nivolumab in 11.4 and 19.7% of SQ and non-SQ NSCLC patients, respectively.

**Table 2:** Overview of treatment-related adverse events

TRAEs	SQ (*N* = 35)	Non-SQ (*N* = 76)	SQ + non-SQ (*N* = 111)
Any grade	24 (68.6)	66 (86.8)	90 (81.1)
Grade 3–4	3 (8.6)	19 (25.0)	22 (19.8)
Serious	2 (5.7)	16 (21.1)	18 (16.2)
Leading to discontinuation	4 (11.4)	15 (19.7)	19 (17.1)

Values are *n* (%).

AE, adverse event; SQ, squamous.

In a pooled analysis, TRAEs that occurred in ≥10% of patients included rash (15.3%), malaise (14.4%), decreased appetite (14.4%), pyrexia (14.4%) and nausea (10.8%). Other TRAEs in ≥5% of patients overall are listed in Table 3. The only grade 3–4 TRAE that occurred in ≥2% of patients was lymphocyte count decreased (4.5%). New TRAEs that were reported after the 3-year follow-up included grade 2 adrenal insufficiency, grade 2 diabetes mellitus, and grades 1 and 3 rash (one patient each) in non-SQ NSCLC patients; there were none in SQ NSCLC patients. New TRAEs resulting in the discontinuation of nivolumab that occurred after the 3-year follow-up included grade 2 adrenal insufficiency and grade 3 rash (one patient each) in non-SQ NSCLC patients and grade 2 fatigue in one SQ NSCLC patient. There were no new cases of pneumonitis or interstitial lung disease from 1 year after starting nivolumab.

**Table 3:** Treatment-related adverse events in ≥5% of patients overall (*N* = 111)

	Any grade	Grade 3–4
Total	90 (81.1)	22 (19.8)
Rash	17 (15.3)	1 (0.9)
Malaise	16 (14.4)	0
Decreased appetite	16 (14.4)	1 (0.9)
Pyrexia	16 (14.4)	0
Nausea	12 (10.8)	0
Pruritus	10 (9.0)	1 (0.9)
Lymphocyte count decreased	10 (9.0)	5 (4.5)
Fatigue	10 (9.0)	1 (0.9)
Diarrhoea	9 (8.1)	0
Arthralgia	7 (6.3)	0
Hypothyroidism	7 (6.3)	0
Rash maculopapular	7 (6.3)	0
Constipation	7 (6.3)	0
Dermatitis acneiform	6 (5.4)	0

Values are *n* (%).

## Discussion

The ONO-4538-05 and ONO-4538-06 phase II studies were performed to examine the efficacy and safety of nivolumab in Japanese SQ NSCLC and non-SQ NSCLC patients. Here, we report the results of the long-term OS and PFS outcomes after ≥5 years of follow-up. Responses to the established conventional therapies are generally poor, as illustrated in CheckMate 017 and 057, in which the 5-year OS and PFS rates for docetaxel-treated patients were just 3 and 0%, respectively (14). Of note, we found that the 5-year survival rate was 14.3% among SQ patients and 19.4% among non-SQ patients, with a pooled rate of 17.8%, and that six patients were still on nivolumab at their last follow-up, >5 years after starting treatment. The 5-year survival rates in these Japanese patients are similar to those reported in prior studies of previously treated NSCLC patients: 16% in a phase Ib study (15) and 13% in a pooled analysis of CheckMate 017 and CheckMate 057 (14). The Kaplan–Meier curves for PFS seemed to plateau after about 3 years, whereas the curves for OS were close to reaching a plateau. This may suggest that the long-term survivors were near a state of remission following nivolumab administration.

We found no marked differences in characteristics between patients who survived for ≥5 years and non-survivors (Table 1). In the previous 3-year follow-up analysis of the ONO-4538-05 and ONO-4538-06 studies, nivolumab as second-line therapy and wild-type *EGFR* gene status were significantly associated with 3-year OS (13). However, treatment lines and *EGFR* status were not significantly associated with 5-year OS in the present analyses, although the 5-year survival rate was numerically high in patients with wild-type *EGFR*. OS and PFS tended to be better in patients with higher PD-L1 expression, with median OS of 18.6 and 21.1 months in patients with PD-L1 expression of ≥1–<50 and ≥50%, respectively. It was reported that upregulation of PD-L1 might be detrimental to prognosis (14). The pooled OS analysis of CheckMate 017 and CheckMate 057 showed that higher PD-L1 expression levels were associated with a greater OS benefit of nivolumab (15), consistent with our present results. However, some patients with PD-L1 expression of <1% also showed a response to nivolumab, with a median OS of 14.6 months. The 5-year survival rate among patients with PD-L1 expression <1% was 11.8%, which exceeds the value reported for docetaxel-treated patients (3%) in CheckMate 017 and CheckMate 057 (14).

Among the 5-year survivors in the present study, 89.5% showed a response to nivolumab. This is similar to the value of 75% reported for 5-year survivors in the previous phase Ib study (16). These results suggest that response to nivolumab greatly contributes to 5-year survival. The 5-year outcomes were generally similar between SQ and non-SQ NSCLC patients, and are also consistent with those in the phase Ib study (16) and with the 3-year follow-up of CheckMate 017 and CheckMate 057 (17).

In terms of the long-term safety of nivolumab, several TRAEs were reported after the 3-year follow-up. The frequency of TRAEs resulting in discontinuation of nivolumab increased slightly after the 3-year follow-up. However, there were no new safety signals during this period of time and, as in the previous report (13), the safety profile of nivolumab was good. Furthermore, there were no additional cases of pneumonitis or interstitial lung disease.

## Limitations

The main limitation of these phase II studies is the lack of a comparator group. In addition, the number of SQ patients was relatively small compared with the number of non-SQ patients. Nevertheless, their outcomes did not differ materially from those of non-SQ patients.

## Conclusions

In this long-term follow-up of patients with SQ or non-SQ NSCLC enrolled in two phase II studies in Japan, 17.8% survived for ≥5 years after starting nivolumab, with six patients still receiving nivolumab and showing an ongoing response at the last follow-up. Of note, long-term survival was observed even in patients with low PD-L1 expression. In terms of safety, no new safety signals were reported after ≥5 years of follow-up, including in patients treated with nivolumab for ≥5 years.

## Data availability

At this time, individual participant data have not been made available. However, reasonable requests made to the corresponding author for de-identified patient-level data that underlie the results reported in this article will be considered.

## Supplementary Material

ONO-4538-0506_5-year_OS_Supplementary_Figures_hyaa157
