# Supplementary material for: Five-year follow-up results from phase II studies of nivolumab in Japanese patients with previously treated advanced non-small cell lung cancer: pooled analysis of the ONO-4538-05 and ONO-4538-06 studies
Source: Jpn J Clin Oncol. 2020 Oct 6;51(1):106–13. doi: 10.1093/jjco/hyaa157 (PMC7767981; doi:10.1093/jjco/hyaa157)
Supplement: ONO-4538-0506_5-year_OS_Supplementary_Figures_hyaa157 [file ono-4538-0506_5-year_os_supplementary_figures_hyaa157.ppt]

## Slide 1
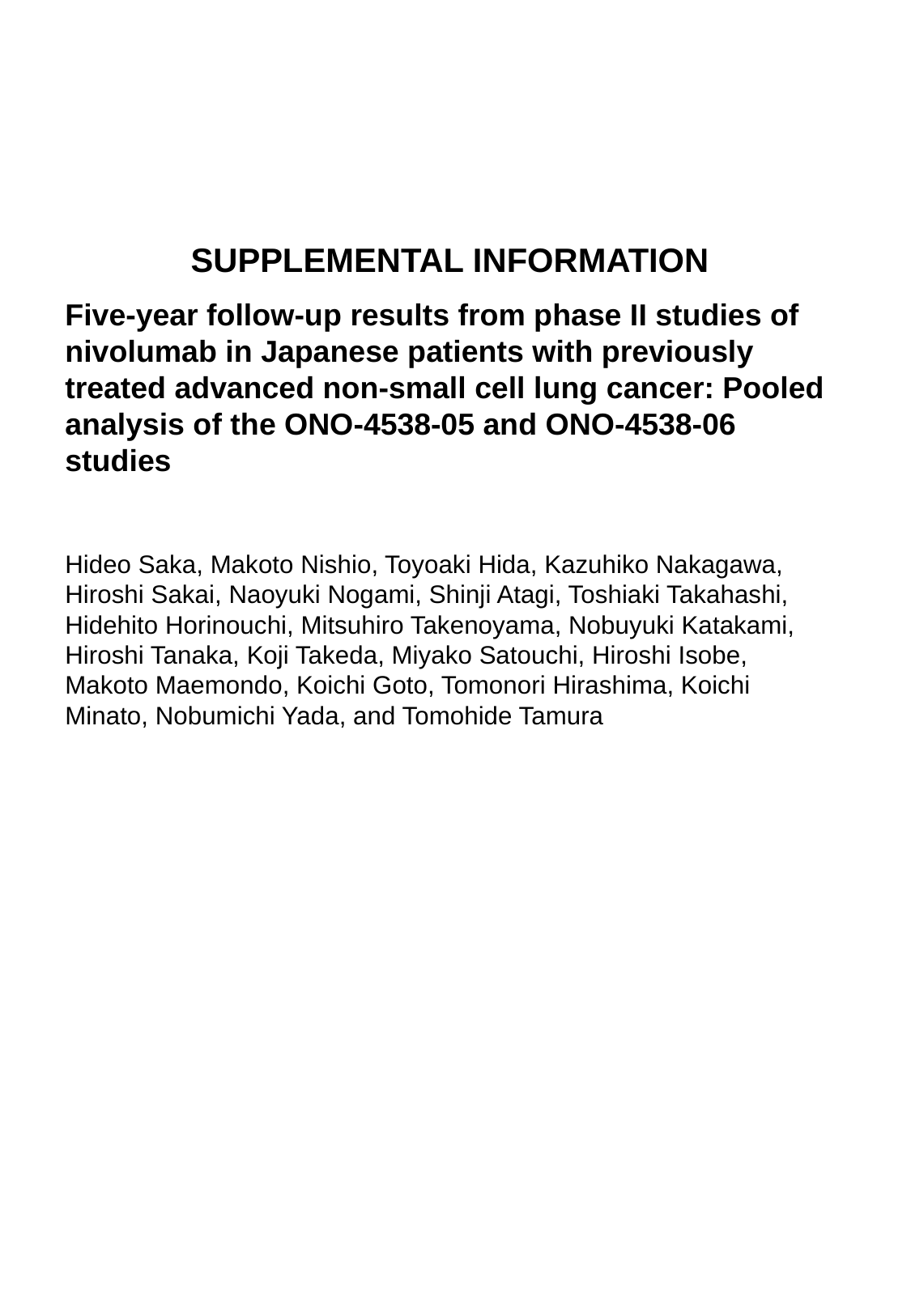

SUPPLEMENTAL INFORMATION
Five‐year follow‐up results from phase II studies of nivolumab in Japanese patients with previously treated advanced non-small cell lung cancer: Pooled analysis of the ONO‐4538‐05 and ONO‐4538‐06 studies
Hideo Saka, Makoto Nishio, Toyoaki Hida, Kazuhiko Nakagawa, Hiroshi Sakai, Naoyuki Nogami, Shinji Atagi, Toshiaki Takahashi, Hidehito Horinouchi, Mitsuhiro Takenoyama, Nobuyuki Katakami, Hiroshi Tanaka, Koji Takeda, Miyako Satouchi, Hiroshi Isobe, Makoto Maemondo, Koichi Goto, Tomonori Hirashima, Koichi Minato, Nobumichi Yada, and Tomohide Tamura

## Slide 2
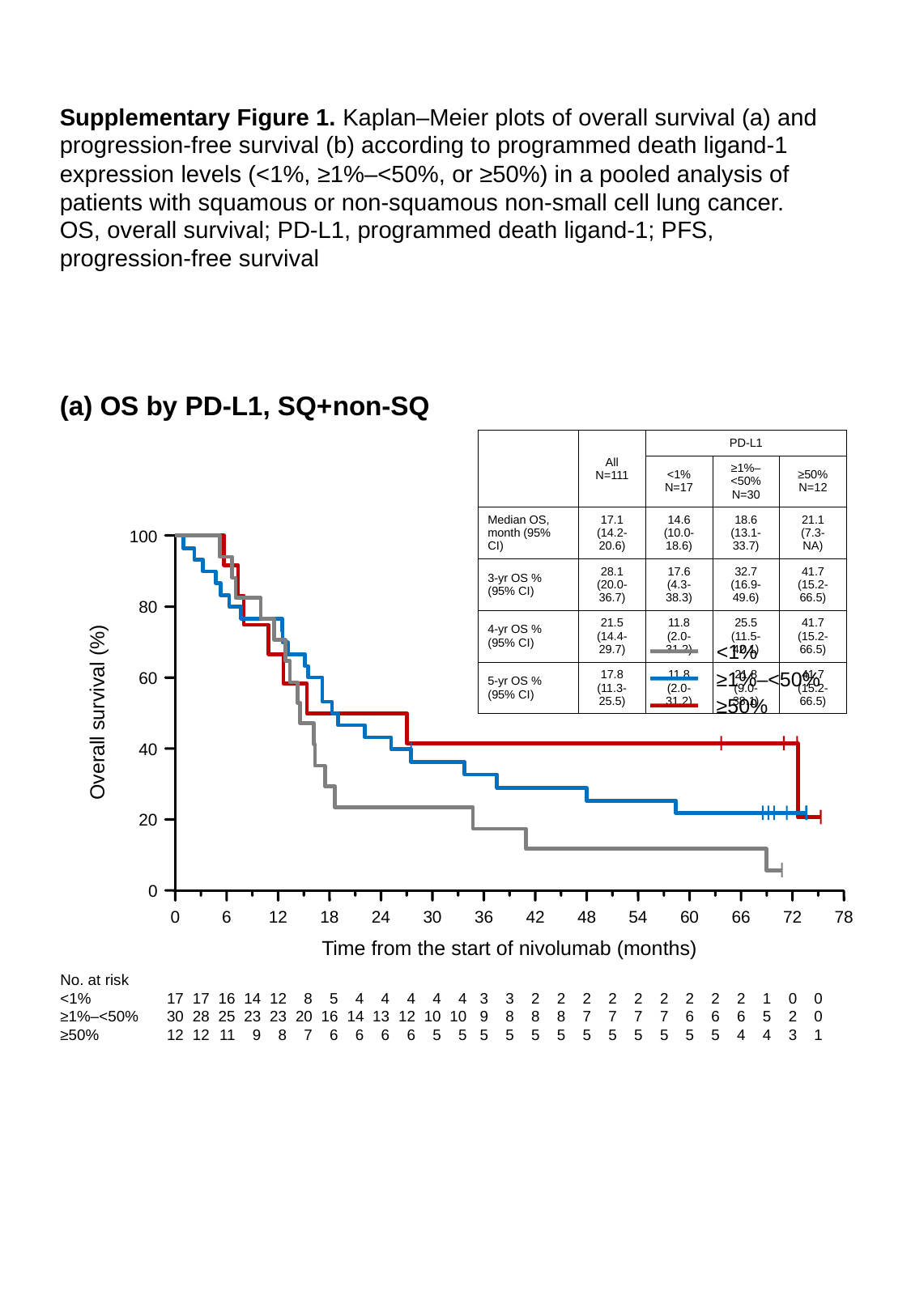

Supplementary Figure 1. Kaplan–Meier plots of overall survival (a) and progression-free survival (b) according to programmed death ligand-1 expression levels (<1%, ≥1%–<50%, or ≥50%) in a pooled analysis of patients with squamous or non-squamous non-small cell lung cancer.
OS, overall survival; PD-L1, programmed death ligand-1; PFS, progression-free survival
# (a) OS by PD-L1, SQ+non-SQ
| | All N=111 | PD-L1 | | |
| --- | --- | --- | --- | --- |
| | | <1% N=17 | ≥1%–<50% N=30 | ≥50% N=12 |
| Median OS, month (95% CI) | 17.1 (14.2-20.6) | 14.6 (10.0-18.6) | 18.6 (13.1-33.7) | 21.1 (7.3-NA) |
| 3-yr OS % (95% CI) | 28.1 (20.0-36.7) | 17.6 (4.3-38.3) | 32.7 (16.9-49.6) | 41.7 (15.2-66.5) |
| 4-yr OS % (95% CI) | 21.5 (14.4-29.7) | 11.8 (2.0-31.2) | 25.5 (11.5-42.1) | 41.7 (15.2-66.5) |
| 5-yr OS % (95% CI) | 17.8 (11.3-25.5) | 11.8 (2.0-31.2) | 21.8 (9.0-38.1) | 41.7 (15.2-66.5) |
100
80
<1%
≥1%–<50%
≥50%
60
Overall survival (%)
40
20
0
0
6
12
18
24
30
36
42
48
54
60
66
72
78
Time from the start of nivolumab (months)
No. at risk
<1%
≥1%–<50%
≥50%
17
30
12
17
28
12
16
25
11
14
23
9
12
23
8
8
20
7
5
16
6
4
14
6
4
13
6
4
12
6
4
10
5
4
10
5
3
9
5
3
8
5
2
8
5
2
8
5
2
7
5
2
7
5
2
7
5
2
7
5
2
6
5
2
6
5
2
6
4
1
5
4
0
2
3
0
0
1

## Slide 3
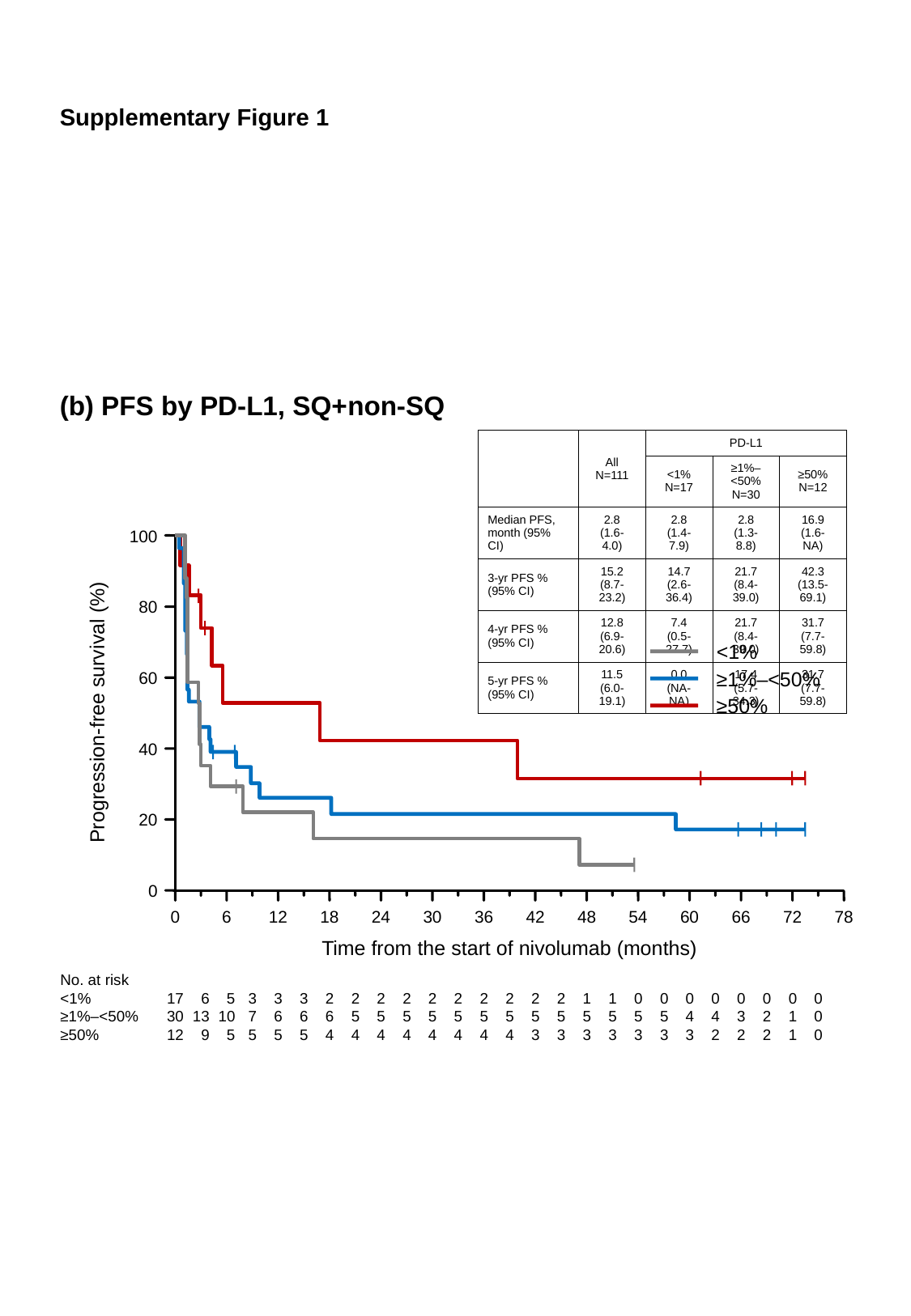

Supplementary Figure 1
(b) PFS by PD-L1, SQ+non-SQ
| | All N=111 | PD-L1 | | |
| --- | --- | --- | --- | --- |
| | | <1% N=17 | ≥1%–<50% N=30 | ≥50% N=12 |
| Median PFS, month (95% CI) | 2.8 (1.6-4.0) | 2.8 (1.4-7.9) | 2.8 (1.3-8.8) | 16.9 (1.6-NA) |
| 3-yr PFS % (95% CI) | 15.2 (8.7-23.2) | 14.7 (2.6-36.4) | 21.7 (8.4-39.0) | 42.3 (13.5-69.1) |
| 4-yr PFS % (95% CI) | 12.8 (6.9-20.6) | 7.4 (0.5-27.7) | 21.7 (8.4-39.0) | 31.7 (7.7-59.8) |
| 5-yr PFS % (95% CI) | 11.5 (6.0-19.1) | 0.0 (NA-NA) | 17.4 (5.7-34.3) | 31.7 (7.7-59.8) |
100
80
<1%
≥1%–<50%
≥50%
60
Progression-free survival (%)
40
20
0
0
6
12
18
24
30
36
42
48
54
60
66
72
78
Time from the start of nivolumab (months)
No. at risk
<1%
≥1%–<50%
≥50%
17
30
12
6
13
9
5
10
5
3
7
5
3
6
5
3
6
5
2
6
4
2
5
4
2
5
4
2
5
4
2
5
4
2
5
4
2
5
4
2
5
4
2
5
3
2
5
3
1
5
3
1
5
3
0
5
3
0
5
3
0
4
3
0
4
2
0
3
2
0
2
2
0
1
1
0
0
0

## Slide 4
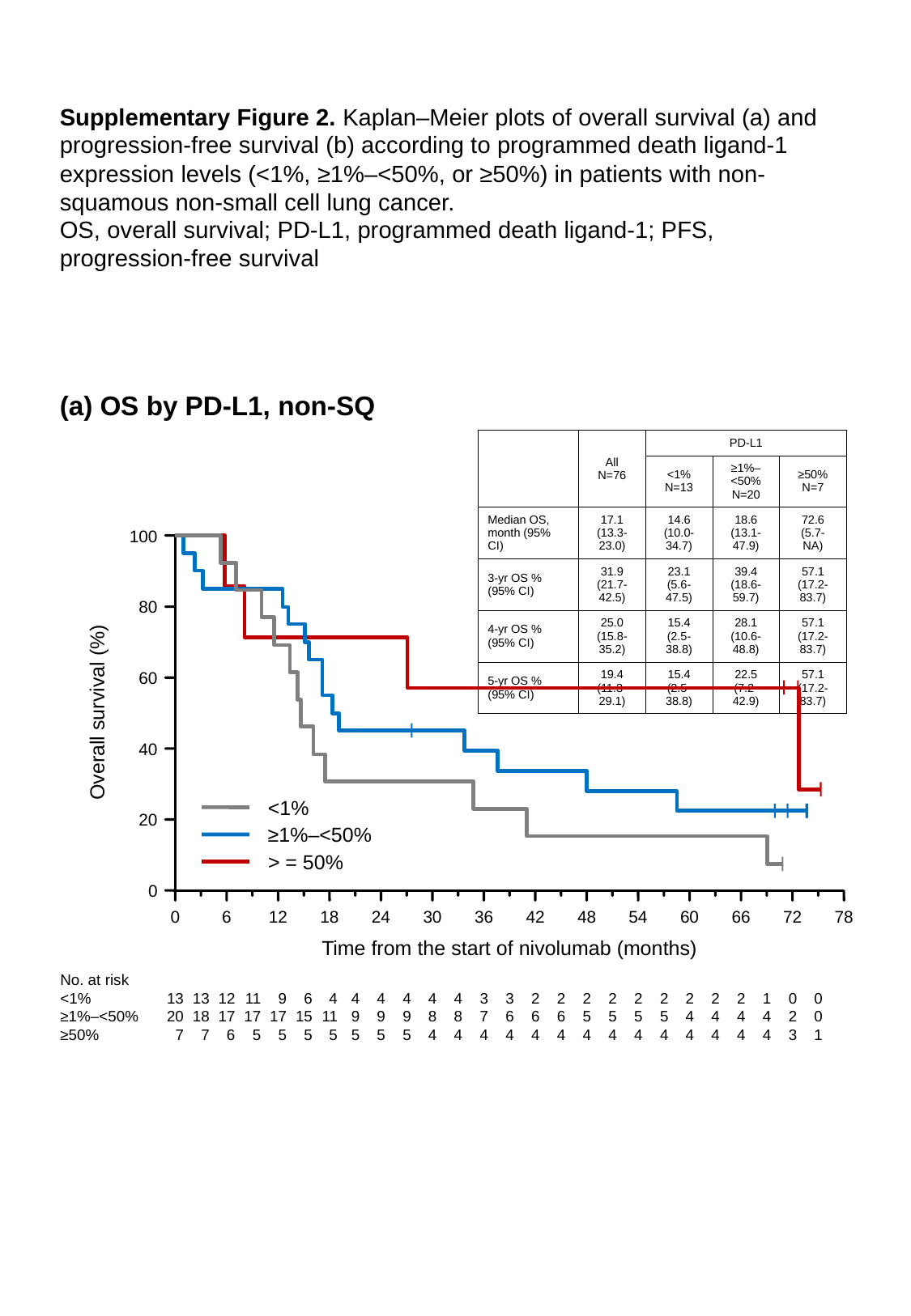

Supplementary Figure 2. Kaplan–Meier plots of overall survival (a) and progression-free survival (b) according to programmed death ligand-1 expression levels (<1%, ≥1%–<50%, or ≥50%) in patients with non-squamous non-small cell lung cancer.
OS, overall survival; PD-L1, programmed death ligand-1; PFS, progression-free survival
# (a) OS by PD-L1, non-SQ
| | All N=76 | PD-L1 | | |
| --- | --- | --- | --- | --- |
| | | <1% N=13 | ≥1%–<50% N=20 | ≥50% N=7 |
| Median OS, month (95% CI) | 17.1 (13.3-23.0) | 14.6 (10.0-34.7) | 18.6 (13.1-47.9) | 72.6 (5.7-NA) |
| 3-yr OS % (95% CI) | 31.9 (21.7-42.5) | 23.1 (5.6-47.5) | 39.4 (18.6-59.7) | 57.1 (17.2-83.7) |
| 4-yr OS % (95% CI) | 25.0 (15.8-35.2) | 15.4 (2.5-38.8) | 28.1 (10.6-48.8) | 57.1 (17.2-83.7) |
| 5-yr OS % (95% CI) | 19.4 (11.3-29.1) | 15.4 (2.5-38.8) | 22.5 (7.2-42.9) | 57.1 (17.2-83.7) |
100
80
60
Overall survival (%)
40
<1%
≥1%–<50%
> = 50%
20
0
0
6
12
18
24
30
36
42
48
54
60
66
72
78
Time from the start of nivolumab (months)
No. at risk
<1%
≥1%–<50%
≥50%
13
20
7
13
18
7
12
17
6
11
17
5
9
17
5
6
15
5
4
11
5
4
9
5
4
9
5
4
9
5
4
8
4
4
8
4
3
7
4
3
6
4
2
6
4
2
6
4
2
5
4
2
5
4
2
5
4
2
5
4
2
4
4
2
4
4
2
4
4
1
4
4
0
2
3
0
0
1

## Slide 5
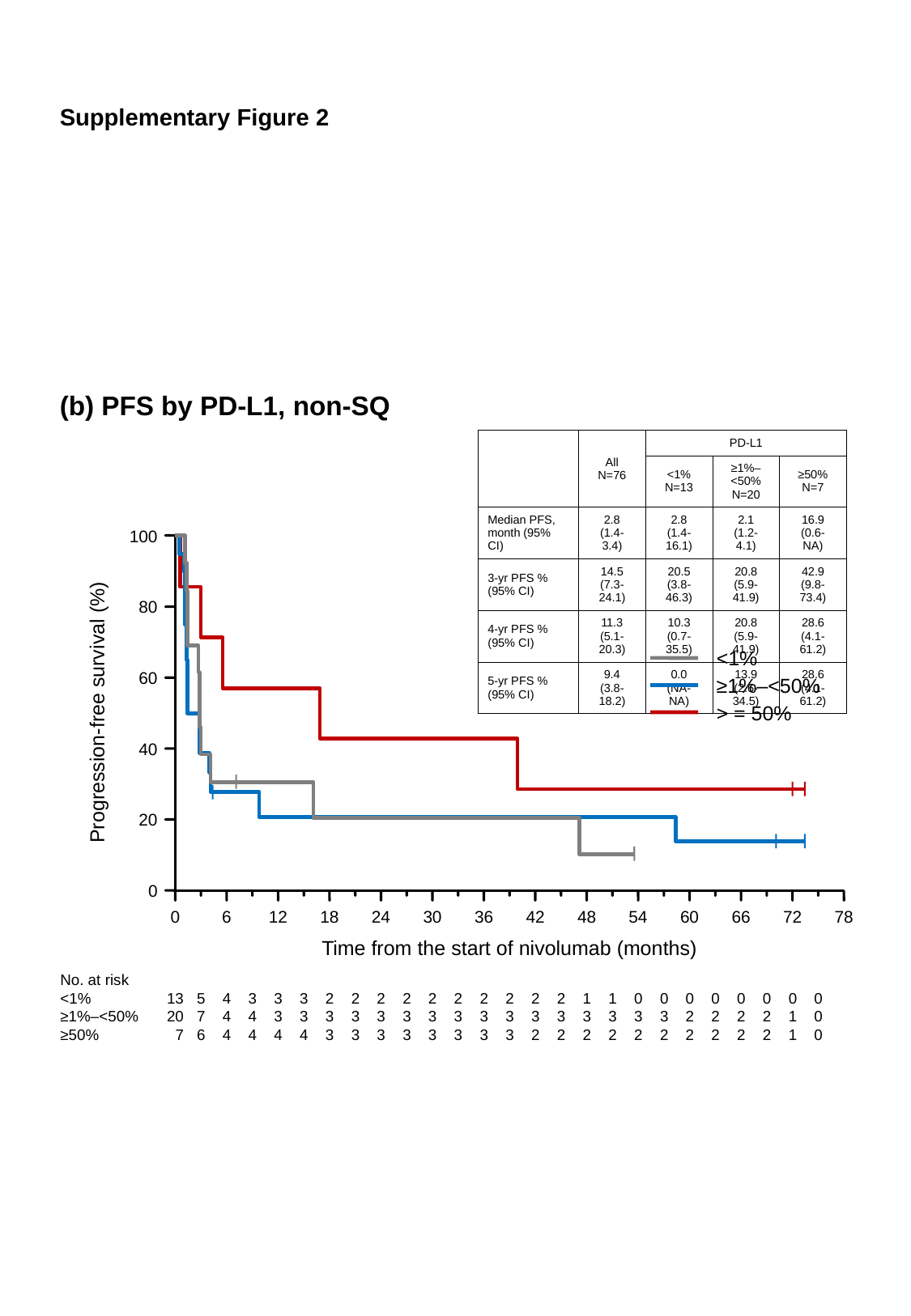

Supplementary Figure 2
# (b) PFS by PD-L1, non-SQ
| | All N=76 | PD-L1 | | |
| --- | --- | --- | --- | --- |
| | | <1% N=13 | ≥1%–<50% N=20 | ≥50% N=7 |
| Median PFS, month (95% CI) | 2.8 (1.4-3.4) | 2.8 (1.4-16.1) | 2.1 (1.2-4.1) | 16.9 (0.6-NA) |
| 3-yr PFS % (95% CI) | 14.5 (7.3-24.1) | 20.5 (3.8-46.3) | 20.8 (5.9-41.9) | 42.9 (9.8-73.4) |
| 4-yr PFS % (95% CI) | 11.3 (5.1-20.3) | 10.3 (0.7-35.5) | 20.8 (5.9-41.9) | 28.6 (4.1-61.2) |
| 5-yr PFS % (95% CI) | 9.4 (3.8-18.2) | 0.0 (NA-NA) | 13.9 (2.6-34.5) | 28.6 (4.1-61.2) |
100
80
<1%
≥1%–<50%
> = 50%
60
Progression-free survival (%)
40
20
0
0
6
12
18
24
30
36
42
48
54
60
66
72
78
Time from the start of nivolumab (months)
No. at risk
<1%
≥1%–<50%
≥50%
13
20
7
5
7
6
4
4
4
3
4
4
3
3
4
3
3
4
2
3
3
2
3
3
2
3
3
2
3
3
2
3
3
2
3
3
2
3
3
2
3
3
2
3
2
2
3
2
1
3
2
1
3
2
0
3
2
0
3
2
0
2
2
0
2
2
0
2
2
0
2
2
0
1
1
0
0
0
